# Are Markers of Allergic Inflammation in Grass Pollen Allergy Influenced by H1 Antihistamines?

**DOI:** 10.3390/jcm11010113

**Published:** 2021-12-26

**Authors:** Ioana Corina Bocsan, Ioana Adriana Muntean, Nicolae Miron, Irena Pintea, Carmen Teodora Dobrican, Corina Ureche, Anca Dana Buzoianu, Diana Deleanu

**Affiliations:** 1 Department of Pharmacology, Toxicology and Clinical Pharmacology, “Iuliu Hatieganu” University of Medicine and Pharmacy, 400012 Cluj-Napoca, Romania; bocsan.corina@umfcluj.ro (I.C.B.); abuzoianu@umfcluj.ro (A.D.B.); 2 Department of Allergology and Immunology, “Iuliu Hatieganu” University of Medicine and Pharmacy, 400012 Cluj-Napoca, Romania; irena.nedelea@umfcluj.ro (I.P.); dobrican.carmen@umfcluj.ro (C.T.D.); diana.deleanu@umfcluj.ro (D.D.); 3 Allergology Department, “Octavian Fodor” Institute of Gastroenterology and Hepatology, 400162 Cluj-Napoca, Romania; 4 Department of Clinical Immunology, Sahlgrenska University Hospital, 41346 Gothenburg, Sweden; nicolae.miron@vgregion.se; 5 First Internal Medical Department, “George Emil Palade” University of Medicine, Pharmacy, Sciences and Technology, 540136 Târgu Mureș, Romania

**Keywords:** soluble adhesion molecules, pollen allergy, eosinophils, antihistamines

## Abstract

Soluble intercellular adhesion molecule-1 (ICAM-1) and soluble vascular adhesion molecule-1 (VCAM-1) play important roles in allergic rhinitis (AR). Treatment with H1 antihistamines improves AR symptoms and in vitro reduces the levels of adhesion molecules. The aim of the study was to evaluate serum levels of ICAM-1 and VCAM-1 in patients with AR to grass pollen and their response to different H1 antihistamines. Material and methods: A total of 50 patients with grass pollen AR were clinically and biologically evaluated. ICAM-1 and VCAM-1 serum levels were evaluated during pollen season before and after treatment with levocetirizine and desloratadine through the ELISA method. Results: ICAM-1, VCAM-1, eosinophils, and total IgE were elevated in patients with AR, compared with healthy subjects. Both antihistamines improved specific symptoms of AR and increased patients’ quality of life during pollen season after one month of treatment. H1 antihistamines reduced VCAM-1, ICAM-1, and total IgE after one-month treatment but not significantly. Patients with increased baseline values tend to remain with increased values after one-month AH1 treatment. Conclusions: ICAM-1 and sVCAM-1 levels are higher in patients with grass pollen-induced AR than healthy controls during pollen exposure. Their serum levels tend to remain at high values during pollen season despite antihistaminic therapy.

## 1. Introduction

Allergic rhinitis is a common disease affecting 20–30% of the general population in industrialized countries. In central Europe, grass pollen is one of the major allergens during late spring and summer, responsible for symptoms of allergic rhinitis accompanied or not by allergic conjunctivitis and/or asthma [1,2,3]. Allergic rhinitis is characterized by an IgE-mediated immune response due to exposure to pollen and several cells and mediators could be identified. After allergen exposure, an early phase of allergic inflammation might occur, releasing immediately specific mediators from mast cells, including histamine. These mediators generate a specific inflammatory response, activating cellular adhesion molecules (CAMs) that are involved in eosinophil’s migration in the nasal mucosa [4,5,6,7]. Vascular cell adhesion molecule 1 (VCAM-1) and intercellular cell adhesion molecule 1 (ICAM-1), which belong to the immunoglobulin superfamily, are expressed on endothelial cells. Adhesion molecules are an important part of the inflammatory network in allergic diseases, involved in persistent inflammation in the upper and lower airways [1,2,3,4,5,6,7,8]. IgE and eosinophils increase during pollen season, because of continuous allergen exposure [9,10,11].

H1 antihistamines (AH1) are the most frequent pharmacological agents used in both intermittent and persistent forms of allergic rhinitis, although more than 50% of the patients did not respond to monotherapy with AH1 [1]. Their anti-allergic effect is related to the blockade of H1 receptors. Research from the last two decades found that the second-generation H1 antihistamines have also an anti-inflammatory effect, decreasing the number of inflammatory cells accumulated in the nasal mucosa and the expression of CAMs [12,13,14,15,16,17,18,19].

The aim of the study was to evaluate the effect of H1 antihistamines during natural exposure to grass pollen and their effects on clinical symptoms, biologic markers, and CAMs. The secondary objective was to identify if there are any differences between 2 commonly used AH1, levocetirizine, and desloratadine.

## 2. Materials and Methods

### 2.1. Study Design

In total, 50 patients with grass pollen allergic rhinitis (median age 27.3 (23–37) years and sex ratio M:F = 1:1) that were evaluated in the Allergology Department, were included in the present study. In addition, 30 healthy volunteers were also included in the control group. The first evaluation was carried out in the middle of grass pollen season in Romania, from 15 May to 15 June (2012–2015). The study protocol was approved by the University of Medicine and Pharmacy “Iuliu Haţieganu” Ethics Committee (Approval No. 535/2 September 2011), and all patients signed the informed consent before enrollment. The study protocol and clinical evaluation were performed according to the initial RCT [19], but only allergic patients to grass pollen were included in the present analysis. The intranasal eosinophils were used as a local marker of inflammation. The exclusion criteria were nasal polyps, acute and chronic upper respiratory infections, other systemic inflammations, autoimmune diseases, cardiovascular diseases, administration of intranasal, inhaled, or systemic corticosteroids or H1 antihistamines within the previous 30 days, and administration of immunosuppressive agents.

### 2.2. Patients’ Clinical Evaluation

Diagnosis of AR was conducted according to the ARIA guideline [1], based on history, typically symptoms at pollen exposure, and skin prick test (SPT). From clinical history, the following demographic data were recorded: age, gender, and living area (rural/urban), symptoms (presence and severity). The severity of AR was assessed using the total symptoms score (TSS) and visual analog scale (VAS). Total symptoms score included rhinorrhea, nasal congestion, sneezing, nasal, and ocular itching, the severity of which symptom was assessed on a scale from 0 (absent) to 3 (severe), retrospectively, for 12 h before presentation. TSS was calculated by adding the score for each symptom. It was considered mild rhinitis if TSS was below 6, while a TSS ≥ 6 represents a moderate-to-severe form of the disease. VAS scale was also evaluated in order to assess the quality of life (QL), VAS value over 6 points meaning patients with a moderate-to-severe form of AR.

After the baseline evaluation, patients were randomly divided into two groups using adaptive biased-coin randomization. In total, 26 patients formed the first groups, and they were treated with levocetirizine 5 mg/day, while the second group of 24 patients received desloratadine 5 mg/day. The treatment was recommended for 4 weeks, until the end of the pollen season in Romania. The second clinical and biological evaluation was performed at the end of the four weeks of treatment.

The presence of asthma symptoms was assessed after 1.5 years, as previously described [19].

### 2.3. Skin Prick Tests (SPTs)

Diagnosis of allergy was established through skin prick test, according to international guidelines [20]. The allergen panel included international recommendations and particularities of exposure to allergens in Romania: *Dermatophagoides pteronyssinus*, *Dermatophagoides farinae*, grass pollens mix (*Agrostis stolonifera, Anthoxanthum odoratum, Dactylis glomerata, Lolium perenne, Arrhenatherum elatius, Festuca rubra, Poapratensis, Secale cereale, Holcus lanatus, Phleum pretense*), cereals pollen, birch pollen, hazel pollen, *Artemisia vulgaris,* and *Ambrosia elatior,* cat and dog dander, and *Alternaria Alternata*. Standardized allergen extracts (Hal Allergy, The Netherlands) were used. SPTs were performed at the beginning of the study.

### 2.4. Biological Evaluation

All the biological parameters were determined before and after 1 month of treatment with H1 antihistamines. The plasmatic level of total IgE was analyzed using the electrochemiluminescence immunoassay method (ECLIA). The obtained values were expressed as UI/mL. A value below <100 UI/mL was considered normal. The intranasal and plasmatic eosinophils (Eos) were manually counted from a slide using May–Grunwald Giemsa staining, and their value was expressed as %. We considered a normal value less than 10% in nasal secretion and less than 4% in the blood.

The serum levels of ICAM-1 and VCAM-1 were determined by the ELISA technique (Quantikinine R&D system, USA). Five ml of blood sample was collected in a tube without anticoagulant and centrifuged within the first hour, followed by serum separation. All the determinations were carried out according to the manufacturers’ instructions.

All blood samples were taken on fasting between 8 a.m. and 11 a.m.

### 2.5. Statistical Analysis

The statistical analysis was performed using SPSS version 21 (Chicago, IL, USA). Data were labeled as nominal, expressed as a percentage, and used continuous variables. The normal distribution for continuous variables was achieved using Kolmogorov–Smirnov test. The influence of different parameters on the evolution of CAM after 1 month was investigated using the ANOVA test for repeating measurements. The Spearman coefficient of correlation was calculated to highlight differences between continuous variables. The level of statistical significance was set at *p* < 0.05.

## 3. Results

Patients’ demographic data are presented in Table 1. There were no statistically significant differences between the two treatment groups.

In patients with AR to grass pollen plasmatic levels of ICAM-1 and VCAM-1 were significantly increased compared with the control group during pollen season (*p* < 0.001, respectively *p* < 0.001). Additionally, total IgE, blood, and intranasal eosinophils were increased at baseline towards healthy volunteers (Table 2).

Genetic predisposition for allergic diseases and asthma was evaluated using the accurate family history of the patients. Overall, 19 patients reported a positive familial history of allergy (asthma, allergic rhinitis, or atopic dermatitis). Patients with a family history of asthma had higher values of inflammatory markers, such as blood eosinophils (median value: 5.5% vs. 9%) than patients with no asthma history, but the group of patients was too small to calculate a statistical significance.

Both investigated H1 antihistamines significantly improved all symptoms of AR and increased patients’ quality of life during pollen season after one month of treatment. TSS significantly decreased after treatment (median 8.5 (5–12) vs. median 4.2 (0–6), *p* = 0.01), with no differences between levocetirizine and desloratadine (*p* = 0.571) (Table 3). A similar reduction was noticed for VAS. The one-month evaluation revealed a reduction in total IgE level (*p* = 0.08), but this was not statistically significant. The reduction in total IgE was not influenced by the type of treatment, patients’ age, sex, living area, or duration of AR (*p* > 0.05).

The same pattern was also observed after a four-week treatment with H1 antihistamines for plasmatic levels of ICAM-1 (*p* = 0.06) and VCAM-1 (*p* = 0.09), compared with basal values, without reaching the level of statistical significance. The reduction in ICAM-1 and VCAM-1 was observed in 42% and 40%, respectively, while in 14 patients (28%), their levels increased despite the treatment.

There was no difference between levocetirizine and desloratadine in the reduction in CAM plasmatic levels. We observed a significant reduction in VCAM-1 and ICAM-1 levels in patients with moderate-to-severe forms, compared with patients with mild rhinitis (*p* = 0.03, *p* = 0.01, respectively). The reduction in CAM levels was not influenced by patients’ age, sex, and type of sensitization. Patients with increased values at baseline tend to remain with increased values after 1-month AH1 treatment (*p* = 0.01).

Intranasal and blood Eo, were significantly reduced after 1-month treatment with AH1 (*p* = 0.03). The reduction in Eo was not influenced by the type of treatment, patients’ age, sex, environment, or duration of AR (*p* > 0.05).

After 1.5 years, 10 patients (20%) had asthma symptoms. The evolution of ICAM-1 and VCAM-1 was also retrospectively assessed in these patients. Nine patients had an increased or stationary evolution of ICAM-1 and VCAM-1 during treatment with H1 antihistamines.

## 4. Discussion

The present study showed a mild anti-inflammatory role of second-generation H1 antihistamines as monotherapy for four weeks of treatment, demonstrated by a reduction in intranasal eosinophils but not on the CAM plasmatic levels in patients with AR to grass pollen during the pollen season. Both desloratadine and levocetirizine improved nasal symptoms and patients’ quality of life if they were administered during pollen season in patients with AR. Both TTS and VAS significantly decreased after treatment, with no differences between the investigated drugs.

AR to grass pollen is characterized by the presence of inflammation in the nasal mucosa during the pollen season under allergen exposure. Allergen exposure induces mast cells degranulation and the release of mediators such as histamine, which are responsible for producing the characteristic symptoms of AR (sneezing, nasal and ocular itching, rhinorrhea, and nasal obstruction) [21,22]. In addition to histamine, other mediators are released from mast cells, such as interleukins 4 and 5 (IL-4, IL-5), leukotriene D4, and E4 (LTD4 and LTE4) [23,24]. Those mediators stimulate infiltration of the nasal mucosa with inflammatory cells, mainly eosinophils, which migrate via CAM in the nasal mucosa [25]. The pattern of chronic allergic inflammatory response is represented by eosinophils infiltration in nasal mucosa [17,26]. Commonly, patients with grass pollen allergy have also other types of sensitization (other types of pollens, house dust mites, molds, animal dander), which may induce also an IgE triggered inflammation outside of grass pollen season, maintaining a minimal specific inflammation in the airway. These cells continue to produce other inflammatory mediators, such as cytokines, chemokines leading to persistent symptoms and tissue damages with structural changes [27,28,29]. Thus, rhinitis persistence and aggravation become more dependent on mediators, which promote infiltration of cells, such as eosinophils and TH2 lymphocytes [21]. AR to grass pollen is a risk factor for asthma development and may appear before or after asthma onset, or during thunderstorm-related asthma [1,2,30,31,32]. In the present study, 20% of the patients developed asthma symptoms within the next 1.5 year follow-up.

Adhesion molecules such as ICAM-1 and VCAM-1 are surface molecules with immunoglobulin-like structure, involved in intercellular adhesion through interaction with the B2 integrin LFA-1. Their importance resides due to cell-to-cell interaction and eosinophil migration in the nasal mucosa in pollen allergic patients [33,34]. In our study, we evaluated the soluble CAM in patients with AR to grass pollen. In grass pollen allergic patients, the inflammation assessed by CAM is higher than in healthy individuals. After the 4-week treatment, H1 antihistamines decreased the plasmatic levels of ICAM-1 compared to basal values, but the reduction was not significant. Similar results were noticed in patients with perennial allergen exposure to house dust mites [35]. The reduction was not significant probably because traces of pollen remained in the atmosphere even if the 4-week evaluation was performed at the end of the pollen season. Grass pollen is the most common pollen encountered in the temperate area where the center is located. The most abundant allergenic grass pollen in many temperate regions originates from tall grasses, such as *Phleum pratense*, *Dactylis glomerata,* and *Arrhenatherum elatius* [36,37]. It is well known the allergenic cross-reactivity between the members of the Pooideae subfamily grasses of temperate regions (*Lolium perenne, Phleum pratense, Poa pratensis*). A study published by a research group from the same country reported an increased level of grass pollen in the air in May, June, and mid of July in some years [38], while the peak of the season for ragweed and mugwort pollens is noticed in late summer and autumn in Romania [39]. The patients were evaluated in the middle of grass pollen in Romania, and according to the previous measurement of pollen level in the air, the symptoms corresponding to allergic inflammation were generated by exposure to grass pollen, not to other pollen allergens that are not encountered in the air in the same period of time.

The reduction in CAMs followed the same pattern as in perennial exposure to house dust mites, as Lee et al previously described [35]. Indeed, the treatment that modified pathological mechanism of disease, including the levels of ICAM-1 and VCAM-1 during pollen season is allergen immunotherapy, not H1 antihistamines, which are more symptomatic and pathogenic therapy than an etiologic one [25]. However, long-term treatment with AH1 might contribute to reducing nasal inflammation through inhibition of cytokines and adhesion molecules production and functions. Treatment with H1 antihistamines for one month in this study during the two-month grass pollen season in Romania may not be sufficient in duration to see a statistically significant change in plasma ICAM and VCAM levels. Patients with increased basal values during pollen season tend to remain with increased values despite AH1 treatment, as long as the allergen exposure persists.

There was no difference between investigated compounds, levocetirizine, and desloratadine in the reduction in CAM plasmatic levels. In the present study, CAMs’ reduction was more significant in patients with moderate-to-severe AR, compared with patients with mild rhinitis. Other studies that investigated the role of AH1 in other forms of AR induced by mite allergy, showed a decrease in mediators released by systemic and intranasal eosinophils after levocetirizine treatment, but CAM levels were not evaluated [34].

Although CAMs are involved in cells migration, including eosinophils to the site of inflammation, the values of intranasal Eo did not correlate with the serum levels of ICAM-1 and VCAM-1 in the present study. Similar to previous studies [40,41], we found that treatment with H1 antihistamines significantly decreased intranasal eosinophils. Due to difficulty obtaining intranasal CAMs, our study focused on plasmatic CAMs as a proxy [27]. Further studies are needed to investigate levels of CAMs in the nasal mucosa and correlate these levels with local infiltration of Eo.

IgE is the primary molecule in the pathogenesis of allergic diseases. Its synthesis and its level are increased after sensitization and it binds to high-affinity Fcɛ1 specific receptors expressed on mast cells. Part of it remains free in the serum and can be determined. Total serum IgE is increased in a variety of diseases, and in allergic subjects may remain also normal. In different clinical studies, IgE levels did not correlate with inflammatory markers such as ICAM-1 or TNF-α values, which are higher in asthmatics but not in those with AR [29]. Usually, IgE also increased during pollen season, and decreases after the season, if the patient is not sensitized to perennial allergens. The bound IgE is responsible for recurrent symptoms and inflammation during the pollen season. In the present study, patients with clinical manifestations induced by grass pollen were included, even if some of them were sensitized to several allergens. The inflammatory response in pollen allergy may differ toward house dust mite allergic patients, due to different aspects of the allergens (perennial, dimensions, enzymatic properties) [42,43,44,45], but this hypothesis needs further investigation. Another reason for the low reduction in total IgE could be related to polysensitization of the patients, even if they did not report clinical manifestation during the grass pollen season.

The main strength of the present study resides in investigating both clinical and pathophysiological effects of two antihistamines in patients with AR, during natural exposure to an elicited allergen. There are also some limitations of this study. Firstly, a small number of patients were included in the study. Secondly, the count of pollens was not performed in the investigated area, and the level of inflammatory markers could not be correlated with the level of exposure. The third limitation resided in the lack of CAM analysis in the nasal secretion, a determination that could not be accomplished due to technical reasons. It might be interesting to correlate the effect of H1 antihistamines on both nasal and blood CAM and eosinophils.

## 5. Conclusions

Patients with AR to grass pollen, during the pollen season, have high intranasal eosinophils levels and high serum levels of ICAM-1 and VCAM-1. H1 antihistamines improve symptoms of AR and reduce intranasal eosinophils. Baseline values of CAMs tend to remain higher during pollen exposure and they were not changed significantly despite AH1 treatment.

## Figures and Tables

**Table 1:** Demographic data.

Parameter		Desloratadine (*n* = 24)	Levocetirizine (*n* = 26)	*p*
Age *		28.05 ± 6.32	29.89 ± 12.17	0.031
Sex ^	male	50% (12)	46.1% (12)	0.263
	female	50% (12)	63.9% (14)	
Living area ^	urban	87.5% (21)	88.5% (23)	0.770
	rural	12.5% (3)	11.5% (3)	
Onset of AR (months) °		24 (6–60)	36 (7.5–68)	0.532
Sensitization ^ to grass pollen	monosensitization	29.2% (7)	23.1% (6)	0.258
	polysensitization	70.8% (17)	76.9% (20)	
Severity ^	mild	25% (6)	23.1% (6)	0.465
	moderate-to-severe	75% (18)	76.9% (20)	
Personal history of allergy		46% (11)	73% (19)	0.5
Familial history of allergy		29.2% (7)	42.3% (11)	0.774

* Data were expressed as mean and standard deviation. ^ Data were expressed as percentage. ° Data were expressed as median and percentiles. Significance *p* < 0.05.

**Table 2:** Nasal and blood eosinophils, plasmatic values of total IgE and adhesion molecules in healthy volunteers and patients with AR.

Parameter	Healthy Volunteers (*n* = 30)	Patients with AR (*n* = 50) Baseline	*p*
Total IgE (UI/l)	<100	255.8 (56.3–599)	<0.001
Nasal Eo (%)	<10	24 (10–46)	<0.001
Blood Eo (%)	<4	8 (2–15)	0.003
ICAM-1 (ng/mL)	111.21 (100–206.3)	235.11 (209.1–276.6)	<0.001
VCAM-1 (ng/mL)	557 (249–891)	996.19 (832.8–1098.2)	<0.001

Significance *p* < 0.05.

**Table 3:** Nasal and blood eosinophils, plasmatic values of total IgE and adhesion molecules initially and after 1 month of AH1 treatment in patients with AR.

Parameter	Patients with AR Baseline (*n* = 50)	Patients with AR after 1 Month-AH1 Treatment (*n* = 50)	*p*
Total IgE (UI/l)	255.8 (56.3–599)	198.7 (49.5–482)	0.08
Nasal Eo (%)	24 (10–46)	18 (10–29)	0.03
Blood Eo (%)	8 (2–15)	5.5 (1–7)	0.03
ICAM-1 (ng/mL)	235.11 (209.1–276.6)	195.42 (124.45–239.89)	0.06
VCAM-1 (ng/mL)	996.19 (832.8–1098.2)	783.19 (689.7–1005.3)	0.09
TSS (score)	8.5 (5–12)	4.2 (0–6)	0.001
VAS (cm)	8.9 (5–10)	3.8 (0–7)	0.001

Significance *p* < 0.05.

## Data Availability

All the records are available at the Department of Allergology and Immunology “Iuliu Hatieganu” University of Medicine and Pharmacy, Cluj-Napoca, Romania.
